# Taxonomic distribution of metabolic functions in bacteria associated with *Trichodesmium* consortia

**DOI:** 10.1128/msystems.00742-23

**Published:** 2023-11-02

**Authors:** Coco Koedooder, Futing Zhang, Siyuan Wang, Subhajit Basu, Sheean T. Haley, Nikola Tolic, Carrie D. Nicora, Tijana Glavina del Rio, Sonya T. Dyhrman, Martha Gledhill, Rene M. Boiteau, Maxim Rubin-Blum, Yeala Shaked

**Affiliations:** 1 The Fredy and Nadine Herrmann Institute of Earth Sciences, Hebrew University of Jerusalem, Jerusalem, Israel; 2 The Interuniversity Institute for Marine Sciences in Eilat, Eilat, Israel; 3 Israel Oceanographic and Limnological Research, Haifa, Israel; 4 Microsensor Research Group, Max Planck Institute for Marine Microbiology, Bremen, Germany; 5 Lamont-Doherty Earth Observatory, Columbia University, New York, USA; 6 Earth and Biological Sciences, Pacific Northwest National Laboratory, Richland, Washington, USA; 7 Joint Genome Institute, Lawrence Berkeley National Laboratory, Berkeley, California, USA; 8 Department of Earth and Environmental Sciences, Columbia University, New York, USA; 9 GEOMAR, Helmholtz Center for Ocean Research, Kiel, Germany; 10 Environmental Molecular Sciences Laboratory, Pacific Northwest National Laboratory, Richland, Washington, USA; 11 College of Earth, Ocean, and Atmospheric Sciences, Oregon State University, Corvallis, Oregon, USA; University of Technology Sydney, Glebe, Australia

**Keywords:** *Trichodesmium*, metagenomes, associated bacteria, interactions, iron, siderophore, siderophore biosynthesis, vitamin B, denitrification, phosphonate, nitrogen fixation

## Abstract

The photosynthetic and diazotrophic cyanobacterium *Trichodesmium* is a key contributor to marine biogeochemical cycles in the subtropical-oligotrophic oceans. *Trichodesmium* form colonies that harbor a distinct microbial community in comparison to the surrounding seawater. The presence of their associated bacteria can expand *Trichodesmium*’s functional potential and is predicted to influence the cycling of carbon, nitrogen, phosphorus, and iron (C, N, P, and Fe). To link the bacteria associated with *Trichodesmium* to key functional traits and elucidate how community structure can influence nutrient cycling, we characterized Red Sea *Trichodesmium* colonies using metagenomics and metaproteomics. Colonies harbored bacteria that typically associate with algae and particles, such as the ubiquitous *Alteromonas macleodii,* but also lineages specific to *Trichodesmium*, such as members from the order Balneolales. The majority of associated bacteria were auxotrophic for different vitamins, indicating their dependency on vitamin production by *Trichodesmium*. The associated bacteria carry functional traits including siderophore biosynthesis, reduced phosphorus metabolism, and denitrification pathways. The analysis supports *Trichodesmium* as an active hotspot for C, N, P, Fe, and vitamin exchange. In turn, *Trichodesmium* may rely on associated bacteria to meet its high Fe demand as several lineages synthesize photolabile siderophores (e.g., vibrioferrin, rhizoferrin, petrobactin) which can enhance the bioavailability of particulate Fe to the entire consortium. Collectively, the results indicate that *Trichodesmium* colonies provide a structure where these interactions can take place. While further studies are required to clarify the exact nature of these interactions, *Trichodesmium*’s reliance on particle and algae-associated bacteria and the observed redundancy of key functional traits likely underpins the resilience of *Trichodesmium* within an ever-changing global environment.

**IMPORTANCE:**

Colonies of the cyanobacteria *Trichodesmium* act as a biological hotspot for the usage and recycling of key resources such as C, N, P, and Fe within an otherwise oligotrophic environment. While *Trichodesmium* colonies are known to interact and support a unique community of algae and particle-associated microbes, our understanding of the taxa that populate these colonies and the gene functions they encode is still limited. Characterizing the taxa and adaptive strategies that influence consortium physiology and its concomitant biogeochemistry is critical in a future ocean predicted to have increasingly resource-depleted regions.

## INTRODUCTION

*Trichodesmium* spp. are a globally relevant group of cyanobacteria that can form surface blooms visible from space (1). Owing to their high abundance and capability to fix both carbon (C) and nitrogen (N), they are often considered key players in the biogeochemical cycling of C and N within the oligotrophic tropical and subtropical oceans (2–5). A key trait underpinning the functionality of *Trichodesmium* is that its filaments can cluster together to form macroscopic colonies several millimeters in size (1–4 mm) (6, 7). There are several proposed benefits to colony formation including a reduction in grazing, facilitating microbial interactions, and enhancing iron (Fe) and phosphorous (P) uptake (8, 9). One particular advantage of colony formation that has received widespread interest within the scientific community is the ability of *Trichodesmium* colonies to coordinate the capture of dust deposited on the ocean surface (10). *Trichodesmium* has subsequently been reported to actively select Fe and P-rich particles and shuffle them to the core of the colony (11–14). Colony formation hereby adds an intriguing spatial component where *Trichodesmium* spp. can act as a biological hotspot not only for C and N but also for the uptake and recycling of key limiting nutrients such as Fe and P.

*Trichodesmium* colonies harbor a unique microbial consortium of epibiotic bacteria. Amplicon sequencing has shown these associated bacteria are distinct from populations in the surrounding oligotrophic surface waters (15–17). At the genetic level, the associated microbial community was found to enrich the total functional potential of the collective colony beyond the traits of N_2_ and C fixation (18–20). By enhancing the genetic repertoire of metabolic functions, or traits, related to the nutrients C, N, Fe, and P, consortium members were predicted to influence the internal cycling of these nutrients (20–23). To understand the biogeochemical contributions of *Trichodesmium* within the global ocean, it is therefore critical to look beyond the metabolic functions present in *Trichodesmium* alone and toward the processes taking place within the larger microbial community. While metagenomic studies of *Trichodesmium* colonies in the Atlantic (19) and the Pacific Oceans (18, 23, 24) highlighted key functional traits within the consortium, only one study linked pathways to the associated bacteria from eight metagenome-assembled bins (19). The assembly of genomes did not represent the majority of sequencing reads, hindering an exploration of traits to their taxonomic affiliation (19). Technological advances in the field of genomics now enable us to revisit and expand on these previous studies. Characterizing how functional traits are distributed across different taxa is critical to understanding the role associated bacteria play in *Trichodesmium* physiology and its concomitant influence over the cycling of key resources such as C and N.

A key functional trait to explore within the *Trichodesmium* consortium through metagenomics is the biosynthesis of Fe-chelating siderophore molecules. Recent evidence from the Red Sea highlights the unique ability of *Trichodesmium* consortium members to acquire particulate Fe from dust through the production of siderophores (12, 13, 22, 25, 26). Siderophore production has been shown to aid the dissolution of particulate Fe from dust, enhancing its bioavailability (27, 28). While *Trichodesmium* is not known to synthesize siderophores (29), studies have suggested the presence of a putative siderophore uptake system in *T. erythraeum* IMS101 (30, 31). *Trichodesmium* is hereby predicted to take advantage of siderophore-producing bacteria to increase the bioavailability of particulate Fe to the colony as a whole (22, 28, 30, 32). In this manner, *Trichodesmium* interacts with its associated bacteria to meet the high Fe requirements needed for the processes of photosynthesis and N_2_ fixation (33). This finding is particularly relevant in light of climate model simulations, which predict an increase in regional dust emissions in areas such as the Red Sea where *Trichodesmium* is known to occur (34, 35). While *Trichodesmium* blooms are a re-occurring phenomenon in this area (36), it represents an under-sampled region where the taxonomic composition of its consortium has not been explored genetically. Furthermore, the presence and taxonomic distribution of siderophore biosynthesis pathways within *Trichodesmium* colonies have not yet been interrogated in depth. Understanding which consortium members positively enhance the bioavailability of Fe within the *Trichodesmium* consortium will further help elucidate key interactions and community dynamics taking place within the colony.

In this study, we conducted an in-depth metagenomic study of *Trichodesmium* colonies collected from the Red Sea. To elucidate how the community structure influences the functional dynamics of the *Trichodesmium* consortium, we identified the different taxa that make up the *Trichodesmium* consortium and examined the presence of functional traits involved in nutrient cycling. We probed our data set for genes and pathways involved in the uptake and metabolism of N, P, Fe, and vitamins. In addition, we contextualize these findings by comparing them to previous metagenomic studies of *Trichodesmium* colonies obtained from the Atlantic and the Pacific oceans. Finally, we analyzed genes of interest to a proteomic data set from *Trichodesmium* colonies collected in parallel.

## RESULTS AND DISCUSSION

A set of 52 non-redundant metagenomic-assembled genomes (MAGs) was obtained from *Trichodesmium* colonies, following quality filtering. Of these MAGs, 42 were of high quality (90% complete and <5% redundancy (Table S1) (37). The 52 MAGs accounted for ~80% of the total sample reads indicating that these MAGs sufficiently captured the diversity of the *Trichodesmium* consortium. The relative abundance of the different MAGs was consistent across the three samples (Fig. S1) with 71% ± 6 of the reads mapping to *T. thiebautii* MAG 52. The remaining MAGs collectively represented the associated bacteria of *T. thiebautii* MAG 52, in this analysis. In parallel to the metagenomic analysis, a proteomic data set matched to 1,253 protein sequences from the 52 different MAGs further probed the activity of *Trichodesmium* colonies within the Red Sea with ±90% of the total 14,074 spectral counts matching to *T. thiebautii* MAG 52 (Table S3).

### *Trichodesmium* colonies host a diverse and flexible consortium of bacteria

**Fig 1:**
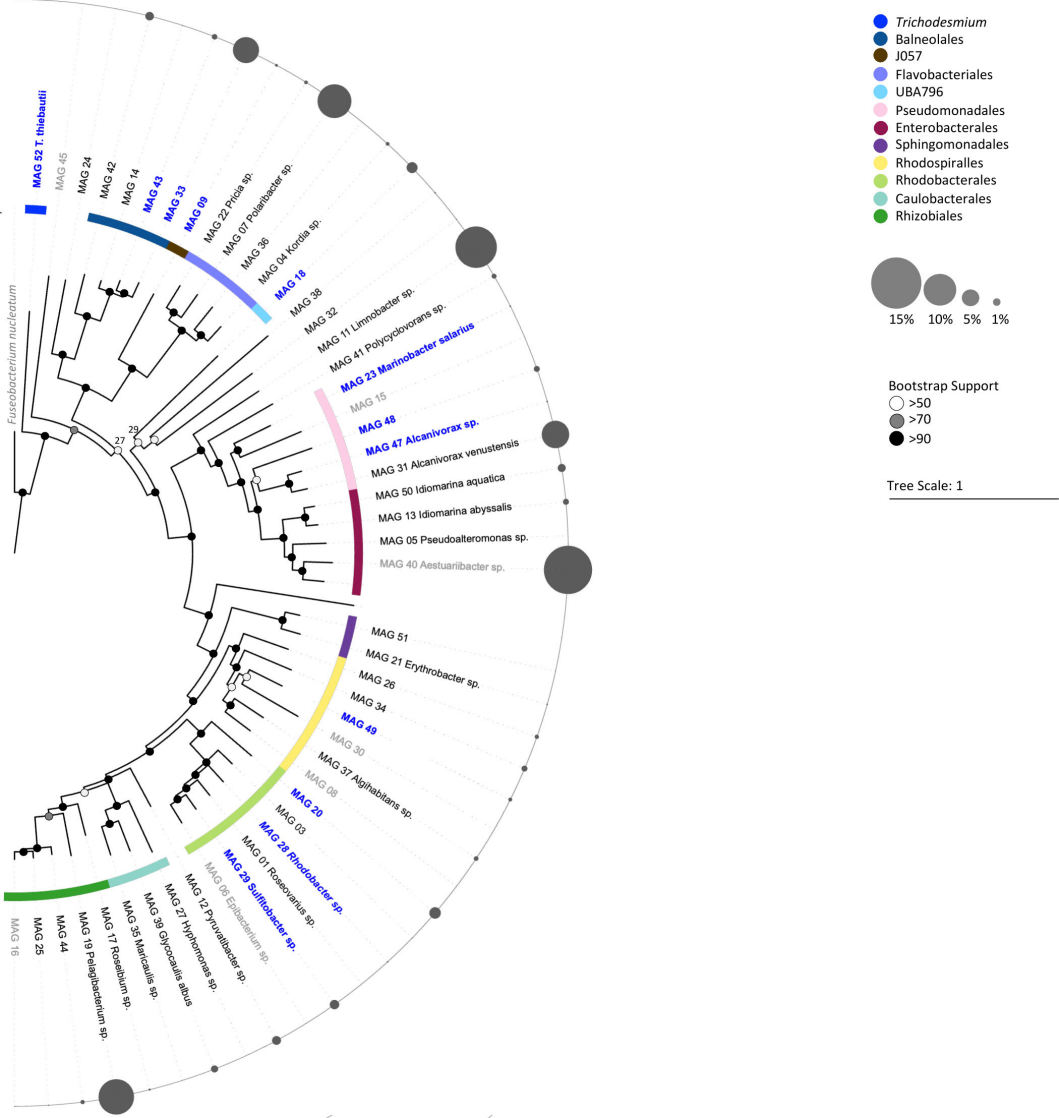
Phylogenetic tree of the 52 MAGs assembled from *Trichodesmium* colonies collected in the Red Sea. The tree is rooted by the outgroup *Fuseobacterium nucleatum*. Known bacterial orders are marked in different colors and several MAGs could taxonomically be identified at the genus level. The relative abundance of read counts mapping to each MAG within the *Trichodesmium* consortium, excluding *Trichodesmium* MAG 52 (±70% of total read counts) is presented as a bubble chart. The MAGs that matched to MAGs assembled from a previous metagenomic data set of Red Sea *Trichodesmium* colonies (38) are highlighted in blue and tentatively represent the core consortium (see Fig. S2). MAGs that did not meet the requirements of a high-quality genome (>90% completeness; <5% redundancy) are indicated in gray. Note that re-occurring MAGs are not necessarily the most abundant MAGs present within the *Trichodesmium* colony (see Tables S1 and S2 for a full taxonomic description of each MAG from each data set).

*Trichodesmium* colonies consisted of a single *Trichodesmium* species (*T. thiebautii* MAG 52) and a diverse consortium of 51 associated bacteria spanning at least 10 different taxonomic orders (Fig. 1). Unlike a previous study (38), our samples did not include any non-diazotrophic *Trichodesmium* populations and the 51 MAGs hereby represent the associated bacteria of *T. thiebautii* MAG 52. The majority of the associated bacterial MAGs belonged to taxonomic orders known to be present in *Trichodesmium* colonies, including Rhodobacterales, Pseudomonadales, Balneolales, Rhodospirillales, Flavobacterales, Enterobacterales, Rhizobiales, and Sphingomonadales (15, 17–19).

We compared our 52 MAGs with MAGs assembled from previous metagenomic data sets of *Trichodesmium* colonies. To allow intercomparisons across data sets, samples were all processed using the same pipeline. In this manner, we were able to assemble an additional 29 MAGs from the Red Sea (38), and 19 MAGs from colonies collected in the Atlantic (19) and the Pacific Ocean (18, 19), respectively (Table S2). Of these MAGs, 11 were common between both Red Sea data sets. As these samples were obtained from two different seasons, these MAGs putatively represent core re-occurring members of the *Trichodesmium* consortium (Fig. S2). These 11 MAGs included typical particle- and algae-associated lineages (39–41) of *Marinobacter* sp. (MAG23; R-02) and *Alcanivorax* sp. (MAG 47; R-15) from the order Pseudomonadales; *Rhodobacter* sp. (MAG 28; R-29) and *Sulfitobacter* sp. (MAG29; R-10) from the order Rhodobacterales. *Alteromonas macleodii* (MAG 10), a ubiquitous bacterium often found in association with phototrophs (42) and previously isolated from *T. erythraeum* IMS101 cultures (43), was also detected in the previous Red Sea data set but did not reach the genome completeness threshold (>75%). *Roseibium aggregatum* (MAG 17), formerly known as *Labrenzia* sp., had previously been identified as a relevant denitrifying bacteria associated with *Trichodesmium* colonies in the Red Sea (44). When comparing our data set to those obtained from the Atlantic, the North and South Pacific oceans (18, 19, 23, 24, 38), we observed taxonomic similarities at the order level rather than at the species level. While most taxonomic orders re-occurred across all data sets, only one Balneolales genome (MAG 33) was present among all *Trichodesmium* metagenomic data sets (T-09; R-03) (Fig. S2). MAGs assembled from these previous data sets accounted for less than 45% of the total sample reads, which may partially explain the lack of common MAGs across ocean basins. Nonetheless, the presence of only a few re-occurring MAGs coupled with the re-occurrence of larger taxonomic groups across all ocean basins highlights the presence of a flexible assemblage of associated bacteria typically found in association with particles and algae. These observed characteristics may have important implications for the community structure and functioning of the *Trichodesmium* consortium.

### *Trichodesmium*-associated bacteria synthesize diverse photolabile siderophores which may increase iron availability from dust

**Fig 2:**
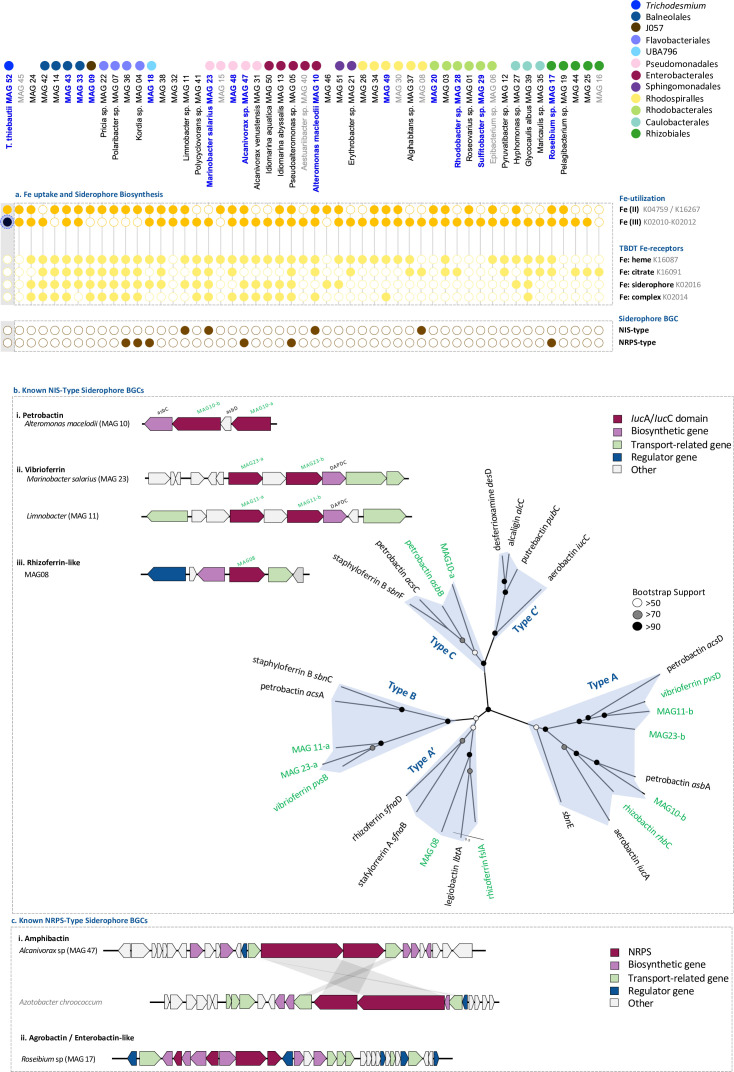
Summary of Fe-uptake and siderophore biosynthesis genes present in MAGs of the *Trichodesmium* consortium (see Table S4 and Materials and Methods for more details). Genes of interest, with a peptide in our proteomic data set are highlighted in black, indicating the protein was present at the time of sampling (see Table S3 for more details). (**a**) *T. thiebautii* MAG 52 does not contain a wide diversity of Fe-uptake genes, and the majority of associated bacteria MAGs contain an uptake receptor for organic Fe:complexes (heme, citrate, and siderophores). Several MAGs of associated bacteria contained non-ribosomal peptide synthetase (NRPS) or NRPS independent synthetase (NIS)-type siderophore biosynthetic gene cluster (BGC) pathways. (**b**) Siderophore BGCs of known NIS-type siderophore BGCs can be clustered according to the presence of one or multiple iucA/iucC domains which enable one to target the closest siderophore BGC match (46). Using this method, we were able to link these pathways to the synthesis of the photolabile (**i**) petrobactin, (ii) vibrioferrin, and (iii) rhizoferrin. (**c**) Siderophore BGCs of known NRPS siderophores. Using antiSMASH 7.0, which aligns BGCs to known siderophores within the MIBiG database, we were able to link two of the six putative siderophore NRPS-type pathways to (**i**) the membrane-bound amphibactin and (ii) agrobactin/enterobactin (see Fig. S3 for more details on the siderophore biosynthesis pathways).

We explored the different Fe-uptake mechanisms present within the *Trichodesmium* consortium, as N_2_ and C fixation in *Trichodesmium* is often constrained by Fe availability (33, 45). Despite the high Fe requirements of *Trichodesmium* (33)*, T. thiebautii* (MAG 52) only contained genes for the uptake of free ferrous (Fe^+2^) and ferric (Fe^+3^) iron, but not organic Fe, suggesting that it is limited to a narrow range of forms it can take up directly (Fig. 2). By contrast, MAGs of the associated bacteria contained a large diversity of Fe-uptake genes which include the ability to take up organically bound Fe, such as heme, citrate, and siderophores (Fig. 2). The proteome of the *Trichodesmium* consortium further indicated that, at the time of sampling, *Trichodesmium* MAG 52 was actively taking up Fe through the presence of a Fe^+3^ uptake transporter (K02012) and increasing its intracellular Fe-pool through the presence of Fe-storage proteins (K03594; K04047) (Table S3).

The genomes of 10 *Trichodesmium*-associated bacteria spread across six different taxonomic orders encoded several different siderophore biosynthetic gene clusters (BGCs) (Fig. 2). The presence of a siderophore biosynthesis pathway was strain specific and not shared by an entire clade or lineage. Similar to previous studies, *T. thiebautii* MAG 52 did not contain any known siderophore biosynthesis pathways (18, 31). The siderophore BGCs could further be separated into four NRPS independent synthetase (NIS)-type and six non-ribosomal peptide synthetase (NRPS)-type siderophores (46, 47). Based on the presence of one or more *iucA/iucC* domains, the four NIS-type BGCs matched closely to petrobactin (*A. macleodii* MAG 10), vibrioferrin (*Marinobacteri* sp. MAG 23; *Limnobacter* sp. MAG 11), and rhizoferrin (Rhodospirillales MAG 08) biosynthesis pathways (Fig. 2). From the six different NRPS-type siderophores, only two matched to a known siderophore BGC, namely that of the membrane-bound amphibactin (100%) (*Alcanivorax* sp. MAG 47) and one resembling the BGC of the siderophores agrobactin (93%) or enterobactin (83%) (*Roseibium* sp. MAG 18) (Fig. 2). In addition, our results indicate the potential of several novel NRPS-type siderophore biosynthesis pathways (Fig. S3). NRPS-type siderophore BGCs are not as well characterized as NIS-type siderophores BGCs and can be difficult to distinguish from other secondary metabolites such as toxins and antibiotics (48). Analysis of the four NRPS-type siderophore BGCs using AntiSMASH 7.0 showed a putative siderophore receptor (SMCOG1082) of a TonB-dependent transporter (TBDT) indicating that these NRPS BGCs are involved in siderophore biosynthesis. While these four NRPS BGCs could not be linked to any known siderophore BGCs, several unknown metallophores have previously been identified and associated with *Trichodesmium* colonies and we speculate that some may be linked to these uncharacterized NRPS BGCs (25). Culture-based research will likely be required to link these pathways to a coinciding siderophore molecule.

Our results indicate that the *Trichodesmium* consortium in the Red Sea may act together with several photolabile siderophore-producing bacteria to mine Fe from dust. The re-occurring consortium members *Marinobacter* sp. (MAG 23), *Alcanivorax* sp. (MAG 47), and *A. macleodii* (MAG 10) are all able to synthesize siderophores (Fig. 2) whose citrate-moiety results in their photolability (49). *Trichodesmium* resides in the natural illumination of surface waters, where sunlight can reductively disassociate Fe from these siderophore complexes and provide a flux of available dissolved and inorganic Fe (50, 51). This dissolved inorganic Fe can be taken up by Fe^+3^ and/or Fe^+2^ transport mechanisms shown to be present in most consortium members (Fig. 2). *T. thiebautii* MAG 52 contains a Fe^+3^ and Fe^+2^ uptake mechanism but only contains a partial TonB system with no known TBDT or siderophore receptors (Table S4) (31, 52, 53) suggesting that these photolabile siderophores can be made bioavailable to *Trichodesmium* upon reductive disassociation by sunlight (29). If proven, these photolabile siderophores act as a “common good” within the *Trichodesmium* consortia, where only a few members carrying the trait are needed to benefit the entire consortium (54). This contrasts with the uptake mode of photostable siderophores, such as desferrioxamine-B (DFOB), which require a specialized TBDT (55). Similarly, amphibactin is a membrane-bound siderophore (56), which prevents its diffusion in the environment and subsequently, its ability to act as a public good to the *Trichodesmium* consortium. A recent gene-knockout study in an *A. macleodii* strain verified the ability of petrobactin to enhance the bioavailability of particulate Fe (28). Collectively, these findings support the idea that *Trichodesmium* can interact with several consortium members that produce photolabile siderophores to acquire Fe from dust.

The detected siderophore BGCs were identified in ubiquitous and copiotrophic bacteria typically found in association with particles and algae (17, 28), including *M. salarius* (MAG 23), *Alcanivorax venustensis* (MAG 47), and *A. macleodii* (MAG 10) (Fig. 2). Siderophore production within the *Trichodesmium* consortium therefore appears to be linked to a lifestyle associated with a rapidly changing environment rich in N and C, where regulating Fe bioavailability can provide a competitive advantage. An *A. macleodii* strain*,* previously isolated from *T. erythraeum* IMS101 cultures, was shown to benefit from the demise of *Trichodesmium* (43, 57), possibly suggesting a more complex interactive relationship between *Trichodesmium* and siderophore-producing bacteria. In line with particle and algae associations, siderophore production has also been associated with other ecological functions including heavy metal detoxification, oxidative stress response, and intra-specific communication (58) (Fig. S4; Table S4). The former two benefits from siderophore production could be relevant to investigate further considering *Trichodesmium*’s ability to center dust, particularly in the presence or absence of sunlight. Although no siderophore BGCs were present in our protein database, the presence of a few, yet reoccurring siderophore-producing MAGs augments previous measurements of siderophore production within the consortium (22, 25, 26) and reveals additional layers of *Trichodesmium* colonies’ intriguing ability to sequester Fe from dust.

### The *Trichodesmium* consortium is heterogeneous in genes related to the uptake and use of inorganic and organic phosphorous

**Fig 3:**
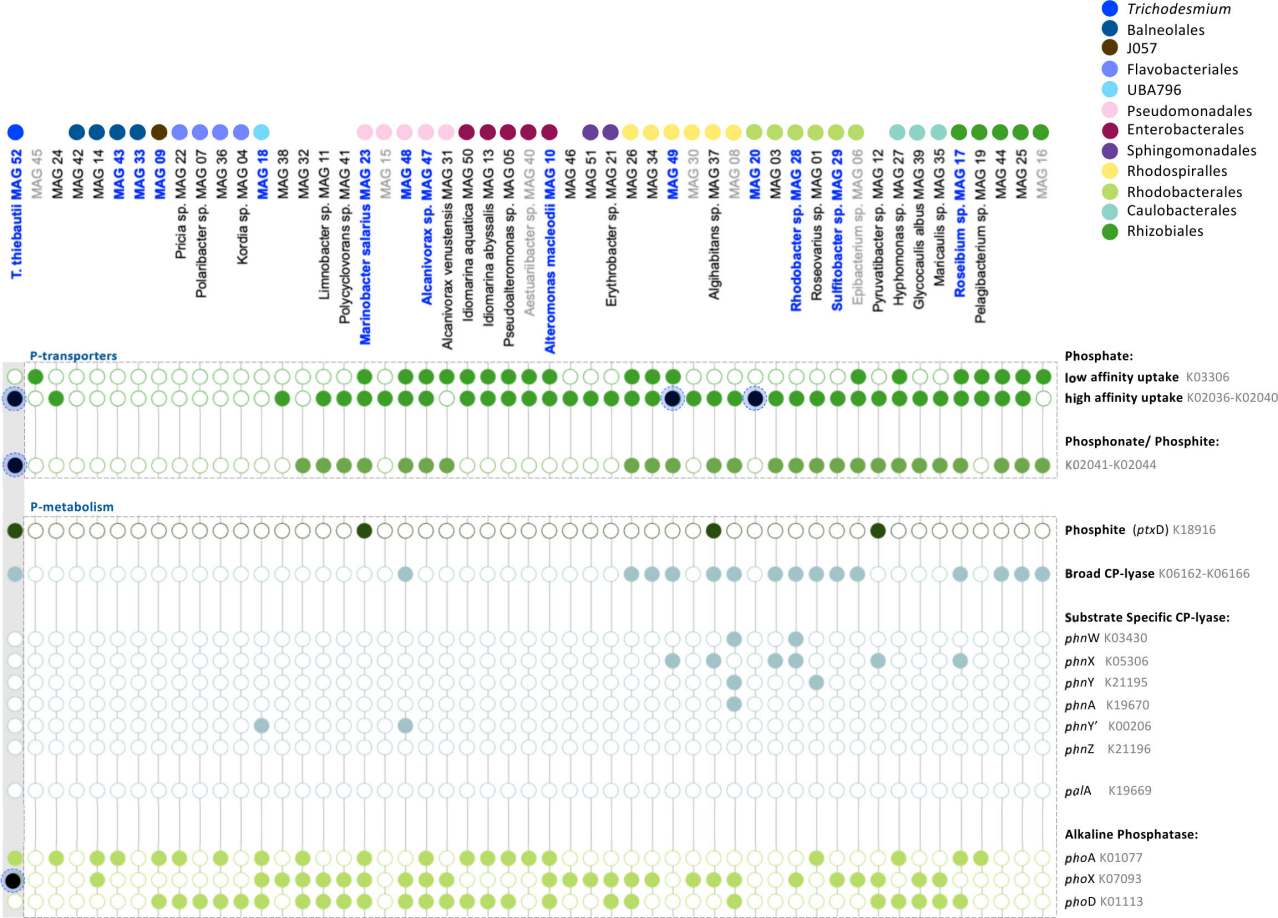
Summary of P-metabolism genes present in MAGs of the *Trichodesmium* consortium (see Table S4 and Materials and Methods for more details). Genes of interest, with a peptide in our proteomic data set are highlighted in black, indicating the protein was present at the time of sampling (see Table S3 for more details). *Trichodesmium* is known to be highly competitive for P, as exemplified by the multitude of (**a**) P-uptake systems for phosphate (*pst*S and *pst*B), phosphonate (*phn*CDE), and phosphonate/phosphite (*phn*CDE/*ptx*ABC). (**b**) Within the *Trichodesmium* consortium, the ability to metabolize a range of phosphonate compounds using a broad specificity C-P lyase (*phn*GHIJKLM) is more prominent than the substrate-specific hydrolysis of 2-aminoethylphosphonate (2-AEP; *phn*WXYZA) or phosphonopyruvate (*pal*A) and phosphite metabolism (*ptx*D).

We predicted that the majority of bacteria associated with *Trichodesmium* has the ability to metabolize phosphite or phosphonate. This can offer a key competitive advantage among consortium members in a P-limited environment (24, 59) including the Gulf of Aqaba in the Red Sea (60). Within a low-P environment, dissolved inorganic P is scarce, and bacteria must scavenge phosphate from dissolved organic P to meet their P-requirements (61). *Trichodesmium* is highly efficient at scavenging and utilizing organic-P through the production of alkaline phosphatase (AP), and the uptake of phosphonate and phosphite (62). Multiple studies have shown that *Trichodesmium* colonies in the Atlantic and Pacific Oceans are hotspots for the reduced P-cycling of phosphite or phosphonate (24, 59) suggesting that the majority of consortium members can process reduced-P. Confirming past studies (24, 63), *T. thiebautii* MAG 52 encodes a set of functions to transport and metabolize diverse forms of P (Fig. 3). This includes the presence of a high-affinity phosphate transporter (*pst*SCAB) and a phosphonate/phosphite transporter (*phn*CDE/*pxt*ABC). In addition to these transport systems, genes related to the utilization and metabolism of organic-P included alkaline phosphatase (AP) (*pho*A*, pho*X), and a carbon-phosphorus (C-P) lyase (*phn*GHIJKLM) which hydrolyses a broad range of phosphonate compounds that can then be taken up. *T. thiebautii* MAG 52 also encodes phosphite dehydrogenase (*ptx*D) which catalyzes the oxidation of phosphite to phosphate. The presence of three *phn*D homologs (K02044), *pho*X (K07093), and *pst*B (K02036), within the proteome, suggests *Trichodesmium* was taking up phosphate, phosphonate, and hydrolyzing phosphoester compounds to meet its P-demand (Table S3). Several of these proteins are regulated by P (63), and their detection in the proteome is consistent with intense competition for P in the low P Red Sea.

As KEGG annotation cannot accurately distinguish between a phosphonate (*phn*CDE) and a phosphite (*pxt*ABC) transporter (64, 65), the presence of adjacent P-metabolism genes can further indicate the P-source linked to it (63). Specifically, the presence of phosphite dehydrogenase (*ptxD*) points to phosphite metabolism while a broad specificity C-P lyase (*phnGHIJKLM*) or the substrate-specific hydrolysis of phosphonopyruvate (*pal*A) or 2-aminoethylphosphonate (2-AEP) to acetaldehyde (*phn*WX), acetate (*phn*WYA), or glycine (*phn*Y’Z) can point to the metabolism of phosphonate (66, 67). Our results showed that the presence of a *phn*CDE/*ptx*ABC uptake system was specific to certain taxonomic orders including Pseudomonadales, Rhodospirillales, Rhodobacterales, Caulobacterales, and Rhizobiales (Fig. 3). These taxonomic orders may therefore have a competitive advantage over members that lack them, particularly when faced with low P conditions. Of these 25 MAGs, 16 could be linked to phosphonate metabolism through the presence of a broad specificity C-P lyase. By contrast, only four MAGs spread across different taxonomic orders appeared to be able to process phosphite due to the presence of phosphite dehydrogenase (Fig. 3). Seven MAGs contained genes associated with the substrate-specific hydrolysis of 2-AEP but pathways were not complete, making it difficult to conclude (Fig. 3). While C-P lyases were not found within our proteomic data set, the presence of broad specificity C-P lyase (*phn*GHIJKLM) appears to be the most prevalent strategy for phosphonate utilization among consortium members, in comparison to 2-AEP hydrolysis (Table S3). This finding is in contrast to a meta-analysis of marine metagenomes where the most common phosphonate degradation strategy within the water column was the substrate-specific hydrolysis of 2-AEP (68). While 2-AEP is the most abundant phosphonate source in the marine environment (69), the prevalence of broad specificity C-P lyases may reflect the relevance of different phosphonate compounds to bacteria residing in *Trichodesmium* colonies.

### The *Trichodesmium* consortium contains genes related to multiple N-metabolic pathways including denitrification

**Fig 4:**
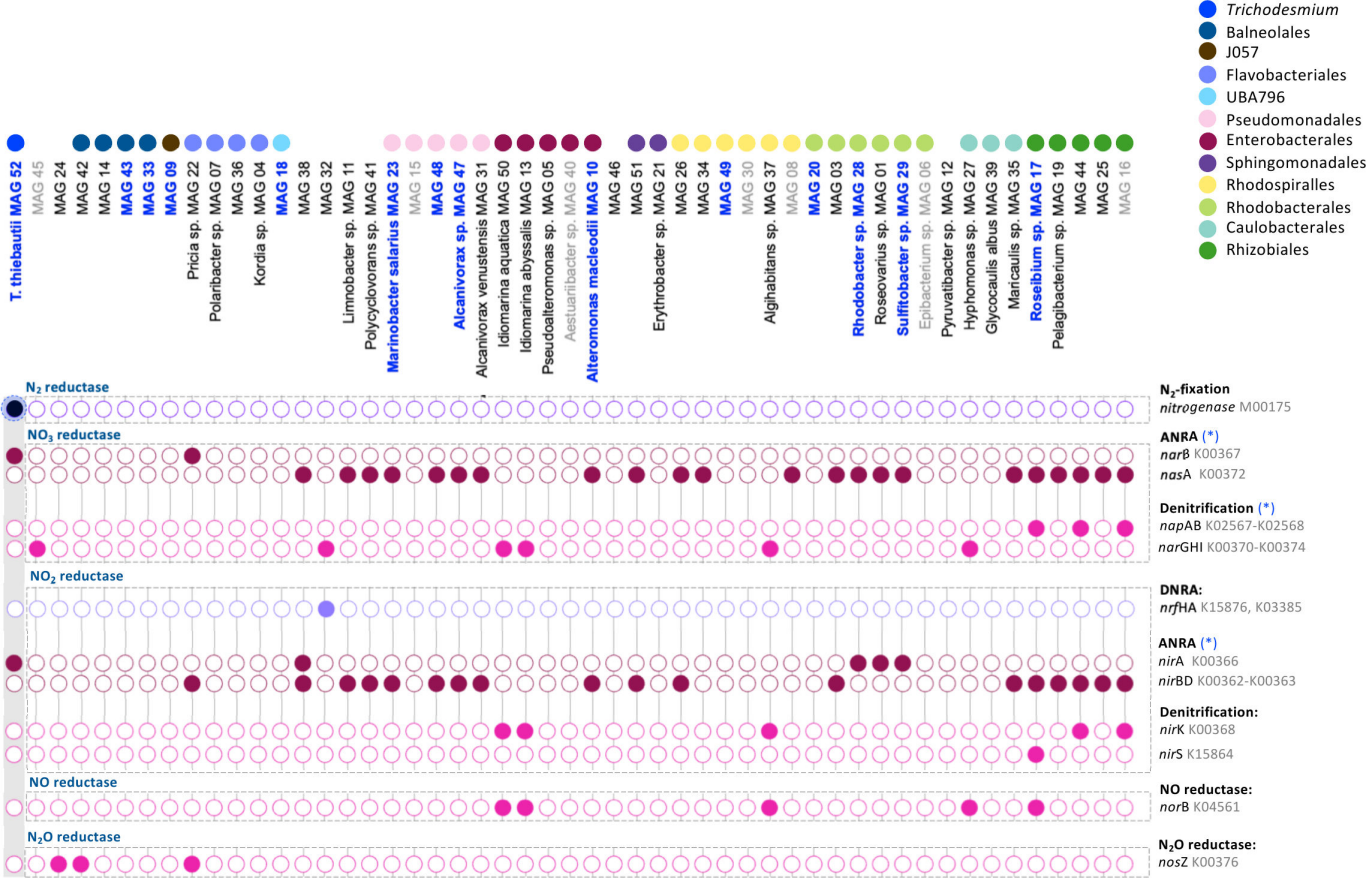
Summary of N-metabolism genes present in MAGs of the *Trichodesmium* consortium (see Table S4 and Materials and Methods for more details). Genes of interest, with a peptide in our proteomic data set are highlighted in black, indicating the protein was present at the time of sampling (see Table S3 for more details). *T. thiebautii* MAG 52 is the only MAG with an N_2_ fixation pathway. When a gene is not considered a marker gene for a specific pathway (and the assignment is hereby putative), the pathway is marked with a blue asterisk. The majority of MAGs contained a putative ANRA pathway to take up nitrate from the environment, while others contained several steps within the pathways of denitrification. Denitrification appears to be modular, where several MAGs completed the denitrification pathway but none contained genes for its entirety.

We explored the presence of multiple N-metabolic pathways predicted to influence the biogeochemical contribution of *Trichodesmium* regarding N_2_ fixation. In *Trichodesmium* colonies sampled from the Red Sea, N_2_ fixation appears to be uniquely attributed to *T. thiebautii* MAG 52 (Fig. 4). This contrasts with a previous study that observed *nif*H genes in both *Trichodesmium* and their associated bacteria (18). In our study, only *T. thiebautii* (MAG 52) contained the full N_2_ fixation pathway (*nif*DKHEB: M00175) and therefore the ability to fix N_2_ within the colony. Similar to other studies in *Trichodesmium* (18, 23, 70), the consortium contained several genes linked to the N-transformation pathways of assimilatory nitrate reduction to ammonium (ANRA), dissimilatory nitrate reduction to ammonium (DNRA), and denitrification (Fig. 4). In the absence of ammonium, the ANRA pathway enables marine bacteria to utilize nitrate as an alternative nitrogen source (71) while denitrification and DNRA are anaerobic respiratory pathways (72). The latter two pathways are N-loss and N-recycling processes, respectively, and are predicted to enable the residing community to conserve and fully utilize N_2_ fixed by *Trichodesmium* (18). Collectively, the N-metabolic pathways present in the consortium can alter the net contribution of *Trichodesmium* colonies to local N-cycling dynamics (21, 72, 73). Nitrate and nitrite reductases are present in all three pathways and the same genes can be associated with multiple pathways in different bacteria (71, 74, 75). Therefore, in addition to KEGG annotations, we linked genes to N-metabolic pathways based on the presence of additional marker genes. Our classification of genes hereby contrasts with previous studies in *Trichodesmium* which relied solely on KEGG annotation (18, 23, 70). In this manner, 19 MAGs containing the nitrite reductase genes *nir*BD could instead be coupled to the nitrate reductase *nas*A, a marker gene for ANRA (71, 74, 75). Contrastingly, previous studies linked the presence of *nir*BD to DNRA within the *Trichodesmium* consortium (18, 23, 70). In our data set, only one MAG could be associated with DNRA using the marker gene *nrf*AH (76). Our functional assignment of genes to N-metabolic pathways hereby remains putative and to confirm whether these reductases are correctly associated with DNRA or ANRA pathways will require a thorough experimental investigation.

Denitrification appeared to be modular within the *Trichodesmium* consortium as different genomes collectively completed the entire pathway (Fig. 4 and 6). Three MAGs contained *nos*Z, the terminal step in denitrification, including *Roseibium aggregatum* (MAG 17), formerly known as *Labrenzia* sp., which was previously identified as an important denitrifying bacteria associated with *Trichodesmium* colonies in the Red Sea (44). While the presence of modularity in denitrification pathways itself is not uncommon (77–79), finding its presence within the *Trichodesmium* consortium indicates the spatial role *Trichodesmium* plays in providing a platform where each step can be performed by a distinct subset of the microbial consortium. The presence of either denitrification or DNRA pathways is rare in the oxic surface ocean, as these processes require anoxic and suboxic conditions (80). In the surface waters, suboxic and anoxic micro-environments were detected in marine aggregates, such as marine snow and fecal pellets, where enhanced microbial respiration results in the consumption of O_2_ and the formation of anoxic microzones (81, 82). *Trichodesmium* colonies may represent an additional environment where such processes can take place within the surface waters of the ocean (44) (83). Our proteomic data set indicated *Trichodesmium* MAG 52 was actively fixing nitrogen (K02588, K02591, and K02586), taking up ammonium (K03320), and metabolizing nitrogen *via* a glutamine synthetase (K01915). Peptides also matched to a glutamine synthetase in consortium members (K01915 and K00982). Peptides, however, did not match any sequences related to ANRA, DNRA, or denitrification pathways (Table S3). While evidence suggests DNRA and denitrification processes are taking place within natural *Trichodesmium* colonies (44, 83), measured O_2_ concentrations in *Trichodesmium* colonies are often well above zero even during darkness (21) and it is still not entirely clear when and where these N-cycling pathways are actively taking place (21, 73). It is suggested that the rapid O_2_ consumption by residing bacteria and the presence of dust (84) could support the formation of suboxic microenvironments in *Trichodesmium* colonies but will require more studies to resolve (73, 84). Altogether, more work is required to fully elucidate the functional potential of consortium members and the exact tradeoffs between interactive members of the *Trichodesmium* consortium regarding N-metabolism.

### *Trichodesmium* consortium members are auxotrophic for vitamins B_1_, B_7_, and B_12_

**Fig 5:**
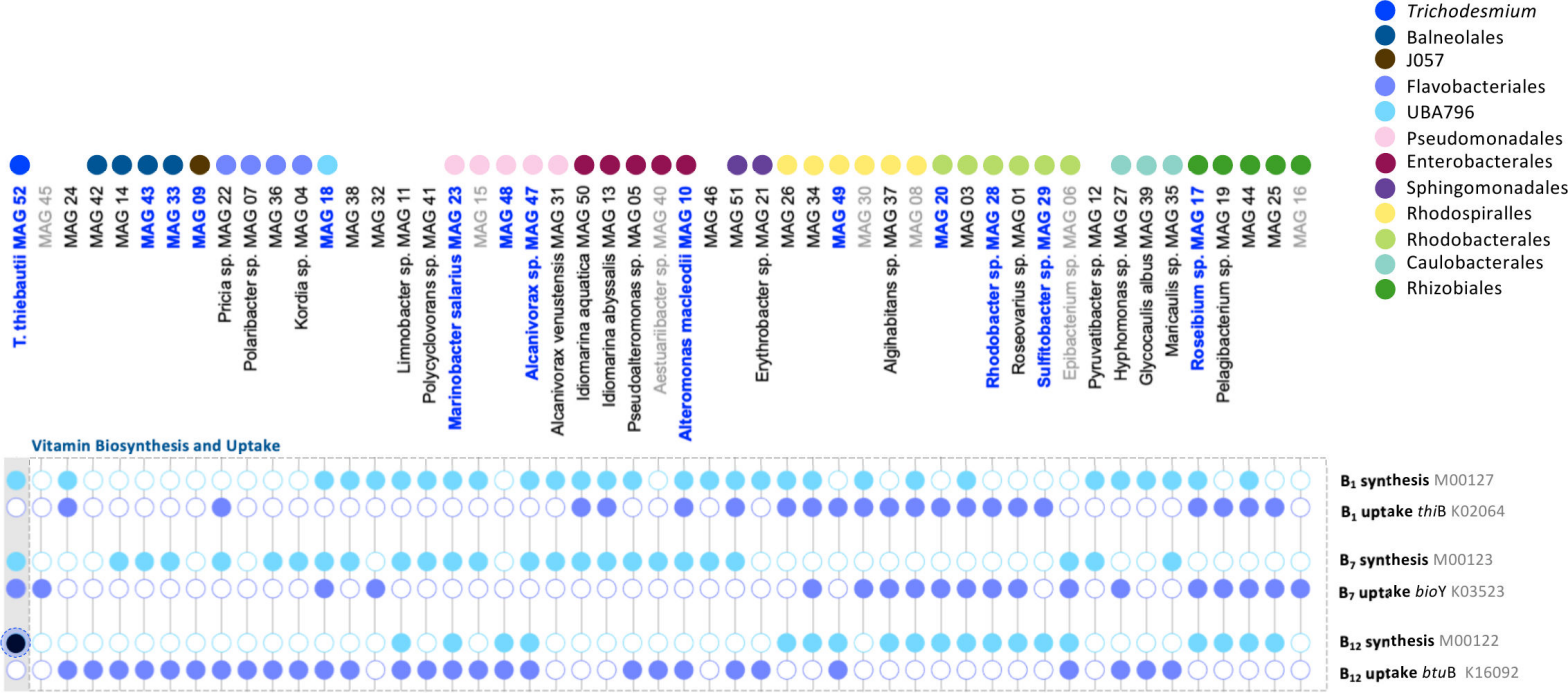
Summary of vitamin B_1_, B_7_, and B_12_ biosynthesis and uptake pathways (see Table S4 and Materials and Methods for more details). Genes of interest, with a peptide in our proteomic data set are highlighted in black, indicating the protein was present at the time of sampling (see Table S3 for more details). *Trichodesmium* contains the biosynthesis pathways for all three vitamins, but the vast majority of associated bacteria were auxotrophic for one or more vitamins. These results suggest that the associated bacteria rely on *Trichodesmium* to obtain their vitamin requirements.

The presence of vitamin auxotrophy within the consortium indicates a dependency of members on *Trichodesmium* for vitamin provision. Our results show that while *Trichodesmium* MAG 52 encodes the biosynthesis pathways for cobalamin (B_12_), biotin (B_7_), and thiamin (B_1_), the majority of associated bacteria (49 of the 52 MAGs) lack a biosynthesis pathway for at least one of these vitamins (Fig. 5). Bacteroidetes, Sphingomonadales, and Enterobacterales species, including *A.s macleodii* (MAG 10), were all auxotrophic for vitamin B_12_. MAGs from the order Rhodospirillales lacked the ability to synthesize vitamin B_7_, whereas Rhizobiales and Rhodobacterales appeared to be auxotrophic for both vitamin B_1_ and B_7_. Only *Pseudoalteromonas* encoded all three biosynthesis pathways, including the re-occurring taxa *Alcanivorax* sp. (MAG 47) and *M. salarius* (MAG 23). These consortium members may also contribute to the synthesis and provision of vitamins B_1_, B_7_, and B_12_ to their auxotrophic counterparts (Fig. 5) but, based on overall relative abundance, *Trichodesmium* represents the most likely source of B vitamins. Previous studies have shown that *Trichodesmium* can synthesize and secrete the vitamin cobalamin (B_12_) to the environment (23). Vitamin uptake genes were not found in our proteomic data set, and only one peptide matched the biosynthesis pathway of vitamin B_12_ (K19221) in *Trichodesmium* (Table S3). Nonetheless, the production and secretion of different vitamins by *Trichodesmium* may be a key determinant of community composition, stimulating vitamin-based interactions among auxotrophic consortium members.

### Nutrient cycling in *Trichodesmium* consortia is likely underpinned by interactions with copiotrophic bacteria

A literature study of the bacterial lineages present within the *Trichodesmium* consortium describes these taxonomic orders as typical primary colonizers of marine surfaces such as algae or sinking particles (39–41). Strains of *Alteromonas*, *Marinobacter,* and *Pseudomonas* often co-occur with phototrophs such as cyanobacteria as well as oil spills, likely as a result of their ability to degrade hydrocarbons and aromatic compounds (42, 85). While the prevalence of these copiotrophic lifestyles within the *Trichodesmium* consortium has been observed in previous studies (17, 19), it is still unclear whether their association with *Trichodesmium* is specific or reflective of a more general interaction between algae or particle associations. Copiotrophy is a widely encompassing term, but it generally concerns opportunistic bacteria with a versatile metabolic capacity to use a wide range of substrates, acting as important remineralizers by rapidly responding toward areas enriched in organic matter (86, 87). It is, therefore, possible to consider the majority of the consortium as active remineralizers of the C, N, P, Fe, and vitamins *Trichodesmium* provides. Viewing *Trichodesmium* as a hotspot for nutrients is supported by our proteomic samples where the most abundant peptides attributed to *T. thiebautii* MAG 52 were related to photosynthetic (K05376, K05377, and K02284) and diazotrophic machinery (K03839, K02588, and K02591) indicating that the processes of N and C fixation were actively taking place at the time of collection (Table S3). Meanwhile, *T. thiebautii* MAG 52 appeared to be limited in Fe and P and actively taking up their various forms through different transporter systems. Due to low peptide biomass, it was more difficult to make conclusions about the protein hits associated with consortium members but included the uptake of nutrients such as amino acids (K09969 and K01999), phosphate (K02040), Fe (K02014), sugars (K02055, K02027, and K10552), and peptides (K02035 and K02032) (Table S3).

In general, copiotrophic bacteria are characterized by the presence of several bacterial-interactive traits (88), indicating the potential of the *Trichodesmium* consortium to form complex interactive networks. The prevalence of copiotrophic bacteria can be supported by the presence of several bacterial-interactive and particle-associated traits (88, 89) (Fig. S4; Table S4), and a previous study has shown gene expression among *Trichodesmium* and associated bacteria appears to be coordinated (23). Although speculative, this could be supported by the presence of several proteins involved in interactive traits such as pilus formation (K02658, K02662, and K02669) and secretion systems (K11003; K03072; K03070, K03110, K03073; K03217) within *T. thiebautii* MAG 52 (Table S3). In this study, 15 associated bacteria MAGs, including re-occurring members from the order Rhodobacterales, contained an acyl homoserine lactone (AHL) biosynthesis pathway (Fig. S4a). Previous studies have shown that the presence of AHLs can modulate N_2_ fixation rates (20) and increase alkaline phosphatase (AP) activity within *Trichodesmium* colonies (90). In particular, consortium members belonging to the phylum Proteobacteria (e.g., Rhodobacterales) are predicted to coordinate their behavior through AHLs (15, 90–92). While our proteomic data set did not indicate their expression at the time of sampling, our results do highlight several potential candidates to further investigate. An AHL biosynthesis pathway was also found in *T. thiebautii* MAG 52. The presence of AHL-homologs had previously been reported for *T. erythraeum* IMS101; however, it lacked an AHL-binding residue suggesting that it is likely not functional (90). Whether *T. thiebautii* MAG 52 contains a functional AHL and can coordinate its gene expression through AHLs is beyond the scope of this analysis. Lastly, the functional association of poorly studied bacterial clades, in particular Balneolales (Table S1), remained more elusive and indicated that we still lack a clear understanding of these bacteria and their interactive role within the *Trichodesmium* consortium. Further investigations will be required to untangle the dynamics of both general and specific interactions taking place between members of the *Trichodesmium* consortium.

### Conclusions and future perspectives

**Fig 6:**
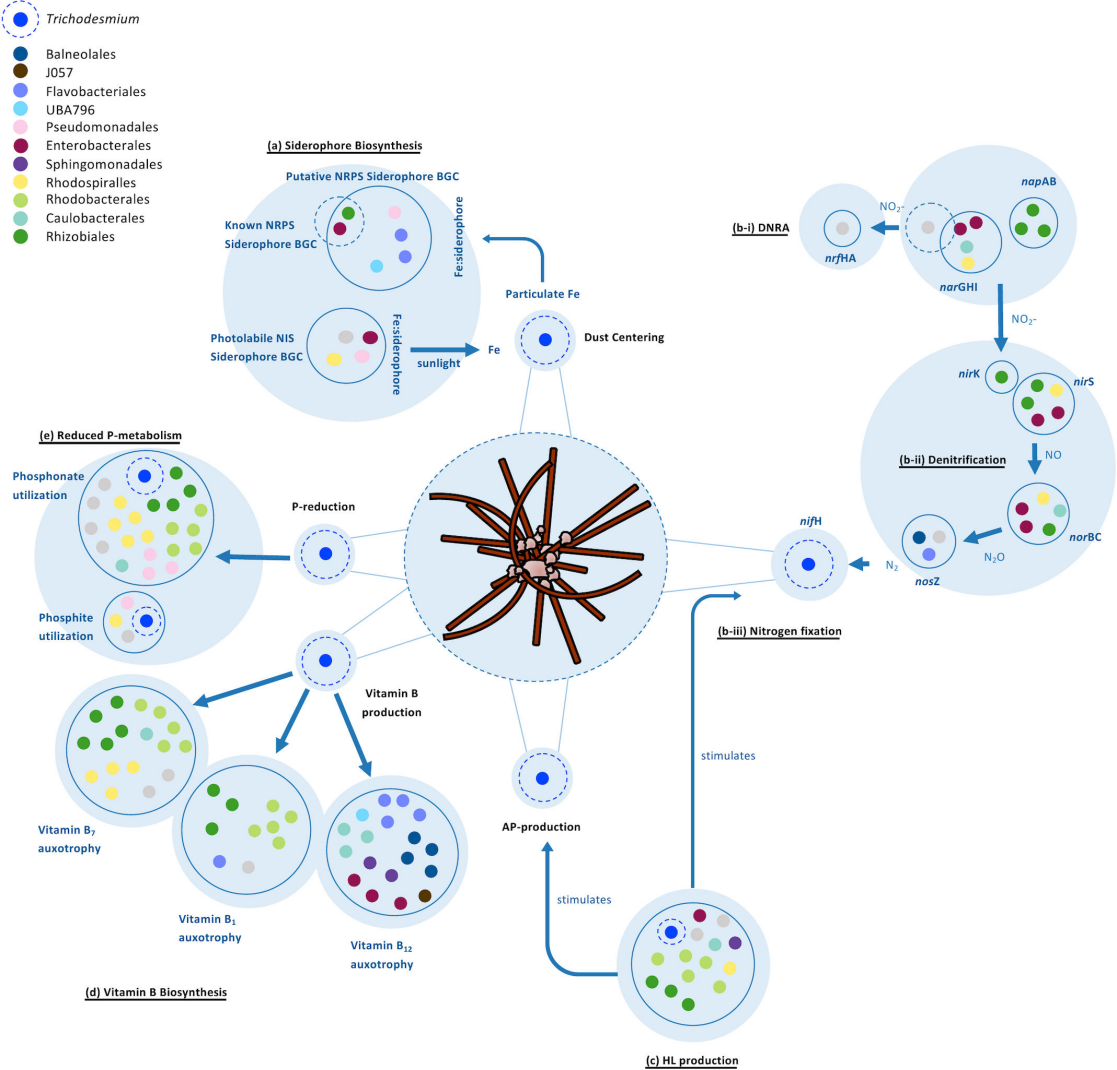
Schematic summary of the potential interactions taking place within the *Trichodesmium* consortia as discussed in this study. Genes for each trait are noted in Table S4. Each blue bubble indicates a different function, and each blue circle pools together the different MAGs containing a particular trait for that function. Each MAG is represented by a colored dot reflective of the taxonomic order it is associated with (gray dots represent MAGs that were not attributed to a specific taxonomic order). *Trichodesmium* is highlighted in cobalt blue, and the important functions *Trichodesmium* displays are highlighted with a dotted circle (see Table S4 for the full list of functional traits, presence/absence, and number of homologs). (**a**) Siderophore biosynthesis. Several siderophore biosynthesis genes are present in a diverse set of MAGs. Siderophores are predicted to mine Fe from dust that has been collected and centered within *Trichodesmium* colonies. Six of the four were identified as known NIS-type siderophores of which petrobactin, vibrioferrin, and rhizoferrin are photolabile. In the presence of sunlight, the Fe complexed to these siderophores disassociates and acts as a public good to the larger consortium. The genes and pathways for (**b-i**) dissimilatory nitrate reduction to ammonium (DNRA) and (**b-ii**) denitrification are present in several MAGs of the consortium. Denitrification appears to be modular within the *Trichodesmium* consortium as a different subset of bacteria contains genes for the terminal step (*nos*Z). (b-iii) *Trichodesmium* MAG 52 was the only MAG containing N_2_ fixation genes in this data set. (**c**) HL production. Several MAGs contain an acyl-homoserine lactone synthesis pathway (AHL), including *Trichodesmium*. AHLs act as an intercommunicating quorum sensing molecule and have been shown to influence the production of alkaline phosphatase (AP) and N_2_ fixation in *Trichodesmium* (associated bacteria can also produce AP but is not depicted here—see Fig. 3). (**d**) *Trichodesmium* can produce the vitamins cobalamin (**B_12_**), thiamin (**B_1_**), and biotin (**B_7_**). Different subsets of MAGs were auxotrophic for different vitamins, defined for each MAG lacking a biosynthesis pathway but containing an uptake pathway for one or more of these vitamins. Very few MAGs are capable of synthesizing all three vitamins and consortium members likely exhibit an interdependent relationship for these vitamins (associated bacteria can also produce vitamins but is not depicted here—see Fig. 5). (**e**) Reduced P-metabolism. *Trichodesmium* colonies have been shown to take up and utilize phosphonate and phosphite in a low-P environment and MAGs containing these pathways may have a competitive advantage over members that lack them.

Our results characterize *Trichodesmium* colonies as a hotspot for C, N, P, Fe, and vitamins in the residing community (Fig. 6). Colonies harbored a flexible assemblage of bacteria, and several core-community members, mostly characterized by a copiotrophic lifestyle adept at processing the nutrients *Trichodesmium* can provide. Vitamins are likely a major component influencing community structure as the majority of bacteria were auxotrophic for one or more vitamins and likely relied on *Trichodesmium* for their synthesis. A key beneficial trait *Trichodesmium* can acquire from its residing community is related to the acquisition of iron from particles through the production of siderophores. *Trichodesmium* harbored several bacteria that can secrete photolabile siderophores, supporting the idea that *Trichodesmium* can meet their high Fe requirements through a collaborative effort to obtain Fe from dust particles collected within the colony. Whether the production of siderophores can further benefit the colony through processes such as heavy metal detoxification and the reduction of oxidative stress may be intriguing to further investigate. To determine the conditions in which such processes may benefit the colony could be explored by moving away from bulk measurements and toward the application of single-colony approaches.

The complex *Trichodesmium* consortium represents an intriguing system to study the intricate interactions taking place within a particle-rich system (Fig. 6), asking how such processes can impact the C, N, P, and Fe cycles in a rapidly changing ocean where shifts in the microbial community composition and their abundance are expected to occur (35, 93). Understanding the factors that drive its community structure across temporal and spatial scales remains an important avenue to continue to explore through a combination of transcriptomic and proteomic techniques. The observed redundancy of multiple traits involved in nutrient cycling among *Trichodesmium* consortium members, including the production of siderophores, indicates the flexibility of a colony while maintaining such traits within the community. Altogether, our results highlight the presence of a dynamic consortium where functional traits are well conserved within the *Trichodesmium* consortium, underpinning the resilience of the colony within an ever-changing ocean.

## MATERIALS AND METHODS

### *Trichodesmium* metagenomic sampling and extraction

*Trichodesmium* puff colonies, 1–2 mm in size, were hand-picked during the spring bloom (6th May 2019) and separated into three samples for metagenomics (~100–200 colonies each) using a 100 µm phytoplankton net at 20 m depth in the Gulf of Aqaba (Eilat, Israel) (29.56°N, 34.95°E). Colonies were washed three times, by gently picking colonies with a pipette and placing them in new petri dishes with fresh 0.2 µm filtered and sterilized seawater, before being filtered on a 0.2-µm, 45-mm polycarbonate (PC) filter and vacuum filtered using a peristaltic vacuum pump. Free-living bacteria are hereby removed while bacteria associated with the *Trichodesmium* colony, both loosely and strongly, are captured and sequenced. This method is also similar to that of other studies (18, 19, 23). Filters were flash-frozen in liquid N_2_ and kept at −80°C. DNA was extracted from the three *Trichodesmium* samples using the DNeasy Plant Pro Kit (Qiagen), with a minor modification to the lysis procedure. The kit-provided tissue disruption tubes were not used. Rather, ∼250 µL zirconia/silica beads (0.5 mm) were added to each sample tube before the addition of Solution CD1, and samples were vortexed for 5 min. The resulting lysate was processed as per the remainder of the manufacturer’s instructions. DNA was quantified fluorometrically on a Qubit 4.0 Fluorometer (Thermo Fisher Scientific, USA). From the three samples, metagenomic libraries were prepared and sequenced with 0.7 billion reads 2  ×  151  bp reads on Illumina NovaSeq S4 at the DOE Joint Genome Institute (California, USA). This study can be found under JGI Gold Study ID number Gs0149370 (https://gold.jgi.doe.gov/study?id=Gs0149370).

### Metagenomic raw read assembly and binning

Sequences were analyzed from this study and compared to previously published metagenomic studies of *Trichodesmium* colonies isolated from the Red Sea (PRJNA804487) (38) from the Pacific (PRJNA435427) (23) (PRJNA358796) (18) and the Atlantic (PRJNA330990) (19) Oceans. All raw sequences, from all data sets, were analyzed using the same protocol described below. The analysis can also be found in the following github depository: https://github.com/cocokoedooder/Trichodesmium_consortium

Metagenomes were assembled, binned, and quantified using the ATLAS (v2) pipeline (94). Briefly, raw sequences underwent quality control through the BBTools suite (95, 96) and were assembled using metaSPAdes (97) (k-mer lengths: 21, 33, 55, 99, and 121 bp). For each sample, MAGs were binned automatically using MetaBAT 2 (98), MaxBin 2.0 (99), and VAMB (100). Using DAS Tool (101) and dRep (102) for quality filtering, a final set of 52 non-redundant MAGs was obtained based on a 97.5% average nucleotide identity (ANI) and 75% completeness cutoff. The 97.5% ANI cutoff was chosen to account for the possibility of multiple *Trichodesmium* sub-species that may be present in our samples, as had been shown in a previous study (38). The completeness and redundancy of each bin were subsequently assessed using CheckM2 v0.1.2 (103). MAGs were considered of high quality if they reached 90% completeness and 10% redundancy and were highlighted throughout our results and figures (37). Genes were predicted using Prodigal (104). In this study, the final set of 52 MAGs did not undergo manual inspection for further MAG refinement. The coverage of each MAG was quantified across each sample by mapping reads back to each MAG. We refer to the MAGs from this study as MAG XX, bacterial MAGs from a previous Red Sea study as R-XX (38), and bacterial MAGs from other metagenomic studies from the Pacific and Atlantic Oceans (18, 19, 23) as T-XX.

### Phylogenetic diversity

MAGs were taxonomically characterized using the genome taxonomy database tool kit GTDB-tk v2.1.1 (105). The taxonomic names of MAGs were classified according to the GTDB taxonomy database of bacterial genomes (version r207.0) where phylogeny is inferred using a concatenated alignment of 120 ubiquitous single-copy proteins (“bac120”) (Tables S1 and S2). The GTDB taxonomic match for each MAG was subsequently linked to its corresponding NCBI taxonomy (https://gtdb.ecogenomic.org/). GTDB taxonomy was preferably used throughout the analysis, but to simplify comparisons within the literature, we used the larger NCBI taxonomic order Rhodospirillales which encompasses the smaller GTDB taxonomic groups of Kiloniellales and Thalassobaculaceae.

Phylogeny was inferred using GTo-Tree (v.1.16.12; default settings) (106) from a concatenation of 74 conserved single-copy HMM markers for bacteria using the best-fit model Q.pfam +R7 model in IQ-Tree (v2.1.3), using the Bayesian information criterion (BIC) (107, 108). Shimodaira-Hasegawa approximate likelihood-ratio test (SH-aLRT) and ultrafast bootstrap approximation (UFBoot) branch support values were estimated from 1,000 bootstraps. The tree was visualized using FigTree (v1.4.4) and rooted using the genome of *Fuseobacterium nucleatum* (PRJNA1419) as an outgroup according to reference (109). MAGs were considered to re-occur in another metagenomic data set if their genome sequence identity was >97.5% similar (Fig. S2).

### Functional diversity

The amino-acid sequence for each MAG was annotated using HMMscan and GhostKoala under default parameters (50% identity cutoff), utilizing the PFAM and KEGG databases, respectively (110, 111). Secondary metabolites (e.g., biosynthesis of siderophores and AHLs) were screened for each MAG using AntiSMASH 7.0. In certain cases, the Rapid Annotation using Subsystem Technology (RAST) server was used to annotate amino-acid sequences using the collection of protein families, FIGfams (112, 113). In addition, each MAG was assessed using METABOLIC (v4.0) which lists the presence of interactive KEGG modules for each MAG (114). Based on these different annotation formats, we inspected each MAG for the presence of functional traits. For each functional pathway or gene, the annotation method, the coinciding KEGG and PFAM ID number, and the number of hits in each MAG can be found in Table S4. Pathways consisting of multiple genes were considered present using a general cutoff of 50% completeness followed by a manual inspection of each pathway for each MAG. For transporter genes, marker genes of substrate-specific receptors were a determining factor in marking their presence or absence in a genome. The presence and absence of different traits for each MAG were displayed using the Interactive Tree of Life (iTOL) visualization tool (115).

#### Iron metabolism

To explore the relationship between *Trichodesmium* and its associated bacteria for the bioavailability of Fe from dust, we searched MAGs for the KEGG IDs of multiple Fe-uptake pathways. Fe-uptake receptors were separated according to abiotic Fe^+2^ (*feo*B: K04759), Fe^+3^ (*afu*A: K02012), and organic Fe uptake which is further separated into Fe-heme (*hem*R: K16087), Fe-citrate (*fec*A: K16091), and Fe-siderophores (*fev*S: K02016). The presence of a Fe:siderophore receptor K02016 should be taken cautiously as its KEGG annotation does not clearly distinguish between siderophores or vitamin B_12_ as a substrate.

Siderophore biosynthesis pathways within MAGs were identified using a conservative approach that required a combination of both KEGG and HMM annotation for each MAG and the use of AntiSMASH 7.0 and FeGenie tools (116) (Fig. S3). Pathways were separately analyzed for both NRPS-type and NIS-type biosynthesis pathways. NIS-type siderophore biosynthesis pathways are more easily identifiable than the two, and we searched for the presence of the Fe-uptake chelate *iuc*A*/iuc*C domain (PF04183). NIS genes were clustered into different types using known NIS biosynthesis genes as defined and described by Carroll and Moore (46). Briefly, genes were aligned using the online Multiple Alignment Fast Fourier Transformation (MAFFT v7.490; L-INS-i) software (117). A phylogenetic tree was constructed from this alignment using IQtree2 (v2.1.3). The resulting best-fit substitution model Q.pfam + F + R4 was selected using Bayesian information criteria (BIC). Branches were assigned using SH-aLRT and UFBoot branch support values were estimated from 1,000 bootstraps. The resulting consensus tree was visualized using FigTree (v1.4.4).

The identification of NRPS-type siderophore biosynthesis pathways required FeGenie, which utilizes biosynthetic pHMMs, and was used to highlight putative NRPS-type siderophores that were further inspected using AntiSMASH 7.0. In AntiSMASH 7.0, MAGs containing an NRPS pathway coupled with a siderophore receptor (SMCOG1082) of a TBDT within the gene cluster were marked as putative NRPS siderophore producers. These pathways were further compared to known NRPS siderophore pathways using the MiBIG database (118) and the KEGG pathway map for NRPS-type siderophores BGC map01053.

#### Vitamin B_1_, B_7_, and B_12_ biosynthesis and uptake

We assume that a bacterial strain is auxotrophic for thiamin (B_1_), biotin (B_7_), or cobalamin (B_12_) if their coinciding genome lacks a vitamin B_12_ biosynthesis pathway but contains a transporter for its uptake. For each vitamin, we searched MAGs for the KEGG IDs and determined the presence of a vitamin biosynthesis pathway using KEGG modules. The presence of a B_1_ biosynthesis pathway (M00127) was determined by the presence of *thi*CDEGL (K00946; K00788; K03149; K00941; K03147). A vitamin B_1_ uptake system was considered present if the marker gene *thi*B (K02064), encoding a vitamin B_1_ binding subunit of an ABC transporter, was present (119) alongside a coinciding transporter system *thi*PQ (K02063, K02062). The presence of a vitamin B_7_ biosynthesis pathway (M00123) was determined by the presence of *bio*ABDF (K01012; K01935; K00833; K00652). A vitamin B_7_ uptake system was present if the marker gene *bio*Y (K03523), encoding a vitamin B_7_ binding subunit of an ABC transporter (120), was present alongside a coinciding transporter system *bio*NM (K16783 and K16784) or *efc*TA_1_A_2_ (K16785, K16786, and K16787). Finally, the presence of a vitamin B_12_ biosynthesis pathway was determined by the KEGG ID module M00122. A putative vitamin B_12_ uptake system was considered present when the marker gene *btu*B (K16092) was observed (121, 122), which encodes a vitamin B_12_ receptor for a TBDT. In addition, we queried the presence of two *btu*F genes (K06858 and K25034) that encode a vitamin B_12_ binding subunit of the ABC transporter *btu*CDF (K06074 and K06073).

#### Nitrogen metabolism

N-metabolism pathways of MAGs were compiled from a selected set of KEGG ID numbers. MAGs were queried for the presence of genes related to N_2_ fixation, dissimilatory nitrate reduction to ammonium (DNRA), assimilatory nitrate reduction to ammonium (ANRA), and denitrification. The ability to fix N_2_ was determined through the presence of *nif*HKD (M00175: K02588, K02591, K02586, and K00531). Denitrification, ANRA, and DNRA can be separated into several steps. All three pathways involve a nitrate reductase which reduces nitrate to nitrite (*nap*AB: K02567, K02568*; nar*GHI: K00370–K00374; *nar*B: K00367). In ANRA and DNRA, the nitrite is further reduced to ammonia by a nitrite reductase (*nrf*AH: K15876 and K03385*, nir*A: K00366; *nir*BD: K00362 and K00363). In denitrification, the nitrite is further reduced to nitric oxide (*nir*S: K15864*; nir*K: K00368), nitric oxide to nitrous oxide (*nor*BC: K04561 and K02305), and nitrous oxide to N_2_ (*nos*Z: K00376). Classification of genes in KEGG (map00910) is largely based on *Escherichia coli*, which appears to form the exception rather than the rule when linking nitrate reductases to different N-metabolic pathways (71, 123–125). We therefore performed an extensive literature search of marker genes to putatively screen and assign genes in each genome to their respective pathways. In this manner, the genes *nor*B and *nir*A present in *Trichodesmium* MAG 52 could be associated with nitrate assimilation (126). Similarly, the genes *nas*A and *nir*BD in 21 MAGs were associated with the ANRA pathway (71) rather than DNRA as had been done in previous studies (18, 23, 70). The nitrite reductases *nir*K or *nir*S acted as marker genes for the presence of denitrification pathways (124) and enabled us to putatively link the nitrate reductases *nar*GHI and *nap*AB to this pathway instead of DNRA in several genomes (10 MAGs) (125). Similarly, n*rf*A acted as a diagnostic for the presence of DNRA within a genome (76), with only one MAG associated with this pathway.

#### Phosphorus metabolism

The presence of multiple P-metabolic pathways within the *Trichodesmium* consortium was explored. We explored several transporters for high-affinity phosphate (*pst*SCAB: K02036–K02038, K02040), low-affinity phosphate (*pit*A: K03306), and a phosphite/phosphonate transporter (*phn*CDE: K02041; K02042; and K02044). We searched MAGs for KEGG ID numbers for the presence of phosphite dehydrogenase (*ptx*D: K18916), which oxidizes phosphite to phosphate, and the broad specificity C-P lyase (*phn*GHIJKLM: K06162-6 and K05780-1), which hydrolyses phosphonate bonds in organic P. In addition, MAGs were queried for the presence of the substrate-specific phosphonate metabolism enzymes including *phn*A (K19670), *phn*X (K05306), and *phn*W (K03430), and collectively these genes were used to indicate the functional potential for reduced P-metabolism. Lastly, MAGs were examined for the presence of alkaline phosphatases (AP) *pho*A (K01077), *pho*X (K07093), and *pho*D (K01113). AP hydrolyses phosphoesters enabling the metabolism of organic-P.

#### Bacterial and particle interaction traits

Known traits that are indicative of putative bacterial interactions were previously summarized and identified by Zoccarato et al. for marine bacterial genomes (88). In addition to siderophore biosynthesis and vitamin B_1_, B_7_, and B_12_ biosynthesis (see previously), MAGs were probed for the presence of AHL biosynthesis genes using AntiSMASH 7.0. All AHLs, including the AHL present in *Trichodesmium,* were homologs of *lux*R and could further be identified by the presence of the pfam domain PF00765. The presence of auxin efflux genes was determined according to RAST annotation. MAGs were screened for KEGG ID numbers for the presence of the quorum sensing regulation gene *lux*R (K07782) and *bja*R1 (K18098), secretion systems (KEGG map03070), and motility and adhesion genes (KEGG map02020) (Table S4). Traits involved in particle interaction were previously described and summarized by Debeljak et al. (89). In addition to Fe-uptake and siderophore biosynthesis pathways, MAGs were screened for KEGG ID numbers involved in metal-response, metal-efflux, and metal-storage pathways (Table S4).

### *Trichodesmium* proteomic sampling and extraction

In parallel to the metagenomic sampling, 20 puff colonies were picked, in triplicate, for proteomic analysis at the Environmental Molecular Sciences Laboratory (EMSL) at Pacific Northwest National Laboratory according to standardized protocols. Colonies were washed three times with filtered seawater, as described above. Samples were centrifuged, and the resulting pellet was diluted in 200 µL 8 M urea and transferred to 2 mL pre-filled micro-organism lysing mix glass bead tubes and bead beat in a Bead Ruptor Elite Bead Mill Homogenizer (OMNI International, Kennesaw, GA) at a speed of 5.5 for 45 s. After bead beating, the lysate was transferred to a new 4 mL tube and immediately placed in an ice block and spun at 1,000× *g* for 10 min at 4°C. An amount of 200 µL of the lysed samples was transferred into 2 mL centrifuge tubes. A bicinchoninic acid (BCA) assay (Thermo Scientific, MA, USA) was performed to determine protein concentration. Following the assay, 10 mM dithiothreitol was added to the samples and incubated at 60°C for 30 min with constant shaking at 800 rpm. Samples were diluted eightfold for preparation for digestion with 100 mM NH_4_HCO_3_, 1 mM CaCl_2_, and sequencing grade trypsin (Promega, WI) was added to all protein samples at a 1:50 (wt/wt) trypsin-to-protein ratio for 3 h at 37 ˚C, 450 rpm. Digested samples were desalted using a 4-probe-positive pressure Gilson GX-274 ASPEC system (Gilson Inc., WI) with Discovery C18 50 mg/1 mL solid phase extraction tubes (Supelco, MO), using the following protocol: 3 mL of methanol was added for conditioning followed by 3 mL of 0.1% trifluoroacetic acid (TFA) in H_2_O. The samples were then loaded onto each column followed by 4 mL of 95:5: H_2_O:ACN, 0.1% TFA. Samples were eluted with 1 mL 80:20 ACN:H_2_O, 0.1% TFA. The samples were concentrated using a speed vac and a final BCA was performed to determine the peptide concentration and samples were fractionated into 12 fractions for LC-MS/MS analysis.

MS analysis was performed using a Q-Exactive Plus mass spectrometer (Thermo Scientific) outfitted with a homemade nano‐electrospray ionization interface. Electrospray emitters used 150  µm o.d. × 20  µm i.d. chemically etched fused silica (124). The ion transfer tube temperature and spray voltage were 300°C and 2.2 kV, respectively. The data were collected for 120  min following a 10-min delay after completion of sample trapping and start of gradient. FT-MS spectra were acquired from 300 to 1,800  m/z at a resolution of 70  k (AGC target 3e6) and the top 12 FT-HCD-MS/MS spectra were acquired in data‐dependent mode with an isolation window of 1.5 m/z at a resolution of 17.5 k (AGC target 1e5) using a normalized collision energy of 30, dynamic exclusion time of 30 s, and detected charge state of an ion 2 or higher. The resulting proteomic samples from this study were deposited in MassIVE (PXD040628).

### Proteomic analysis

Peptide matching was performed using MSGF+ software (127) against the amino-acid sequences obtained from the matching metagenome data set through PROKKA (128) using the ATLAS pipeline (v2) (94) resulting in 5,292 unique clean peptide sequences that could subsequently be mapped to 1,253 different protein sequences. MSGF+ was used in target/decoy mode with 20 ppm parent ion tolerance, partial tryptic rule, and M-oxidation (+15.9949) as a dynamic modification. Best matches from the MSGF+ searches were filtered at a 1% false discovery rate based on the target/decoy model and this final set of peptides was used in the consequent quantitative analysis. We obtained a total of 14,074 spectral counts without consideration of peptide specificity for proteins and MAGs, to encompass the widest range of putative protein matches. 91.6% of peptides mapped to a unique protein sequence and 92.7% of peptides were found in a single MAG, indicating that for most peptides there was little ambiguity regarding its protein and taxonomic match. Peptide sequences could be linked to 81 proteins that were associated with 65 genes of interest. Their sequences were manually checked for MAG and protein specificity to assess the reliability of these counts. Spectral count normalization was performed using the normalized spectral abundance factor (129). Briefly, counts were divided by the matching peptide length and their relative abundance was calculated from the total number of spectral hits per sample (Table S3).

## Data Availability

Raw sequences are available under the Gold Study ID number Gs0149370 and correspond to Gp0503298 (PRJNA1022442; SAMN37607796), Gp0503299 (PRJNA1022443, SAMN37607797), and Gp0503300 (PRJNA1022444, SAMN37607795). The 52 metagenomic assembled genomes analyzed in this study are available at NCBI under the BioProject number PRJNA944101. Proteomic samples are available at MassIVE under the project accession number MSV000091416. Scripts of the data analysis and figures can be found in the github depository: https://github.com/cocokoedooder/Trichodesmium_consortium

## SUPPLEMENTAL MATERIAL

Figure S1 msystems.00742-23-s0001.pdf Relative abundances of bacterial MAGs from sampled *Trichodesmium* colonies indicate that the associated bacterial distribution is consistent.

Figure S2 msystems.00742-23-s0002.pdf Phylogenetic tree of the 52 MAGs together with MAGs assembled from 3 other *Trichodesmium* metagenomic data sets from colonies collected in the Red Sea, the Pacific Ocean, and the Atlantic Ocean.

Figure S3 msystems.00742-23-s0003.pdf NIS and NRPS-type siderophore biosynthesis pathways identified in MAGs of associated bacteria.

Figure S4 msystems.00742-23-s0004.pdf MAGs were probed for bacterial-interactive and particle-interactive traits.

Supplemental Tables msystems.00742-23-s0005.xlsx Tables S1-S4.

ASM does not own the copyrights to Supplemental Material that may be linked to, or accessed through, an article. The authors have granted ASM a non-exclusive, world-wide license to publish the Supplemental Material files. Please contact the corresponding author directly for reuse.
