# Supplementary material for: Taxonomic distribution of metabolic functions in bacteria associated with Trichodesmium consortia
Source: mSystems. 2023 Nov 2;8(6):e00742-23. doi: 10.1128/msystems.00742-23 (PMC10734445; doi:10.1128/msystems.00742-23)
Supplement: Figure S1 — Relative abundances of bacterial MAGs from sampled Trichodesmium colonies indicate that the associated bacterial distribution is consistent. [file msystems.00742-23-s0001.pdf]

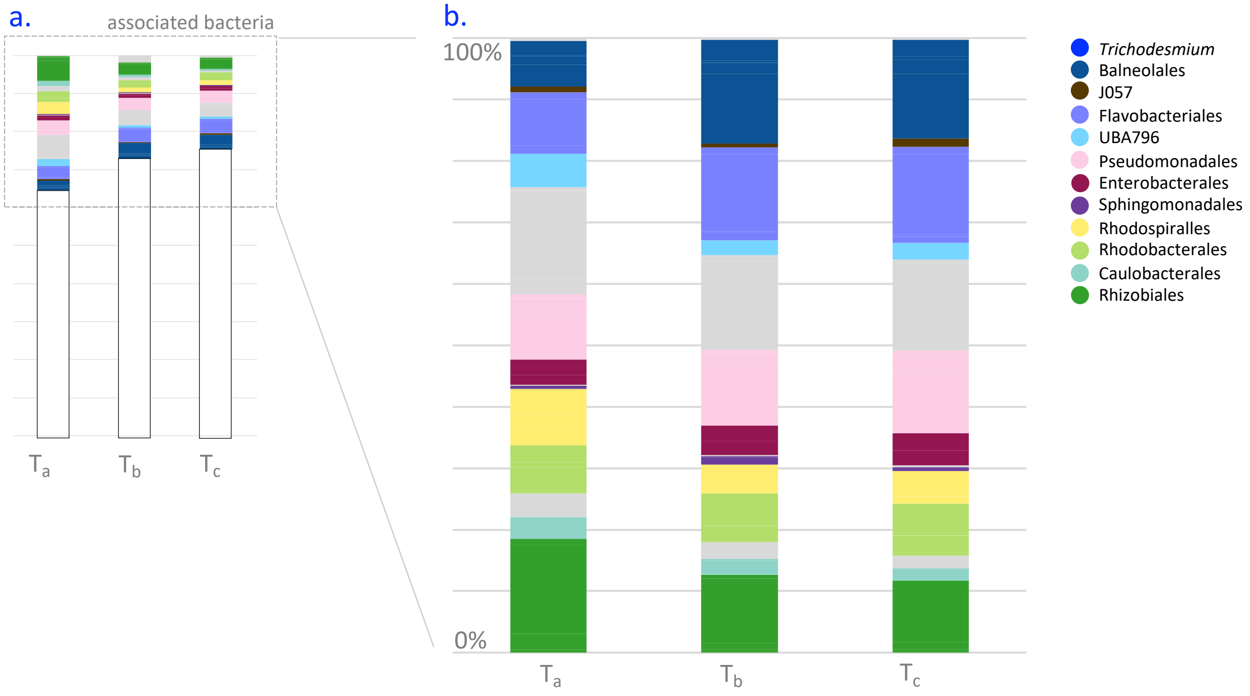

Supplementary Figure 1. Relative abundance of bacterial MAGs from sampled *Trichodesmium* colonies indicate that the distribution of associated bacteria is consistent between samples. The 51 MAGs are stacked, and colors reflect the different bacterial orders each MAG belongs to and 'other' in grey. The relative abundance (%) for each MAG and its standard deviation can be viewed in Supplementary Table 1.
