# Supplementary material for: Taxonomic distribution of metabolic functions in bacteria associated with Trichodesmium consortia
Source: mSystems. 2023 Nov 2;8(6):e00742-23. doi: 10.1128/msystems.00742-23 (PMC10734445; doi:10.1128/msystems.00742-23)
Supplement: Figure S2 — Phylogenetic tree of the 52 MAGs together with MAGs assembled from 3 other Trichodesmium metagenomic data sets from colonies collected in the Red Sea, the Pacific Ocean, and the Atlantic Ocean. [file msystems.00742-23-s0002.pdf]

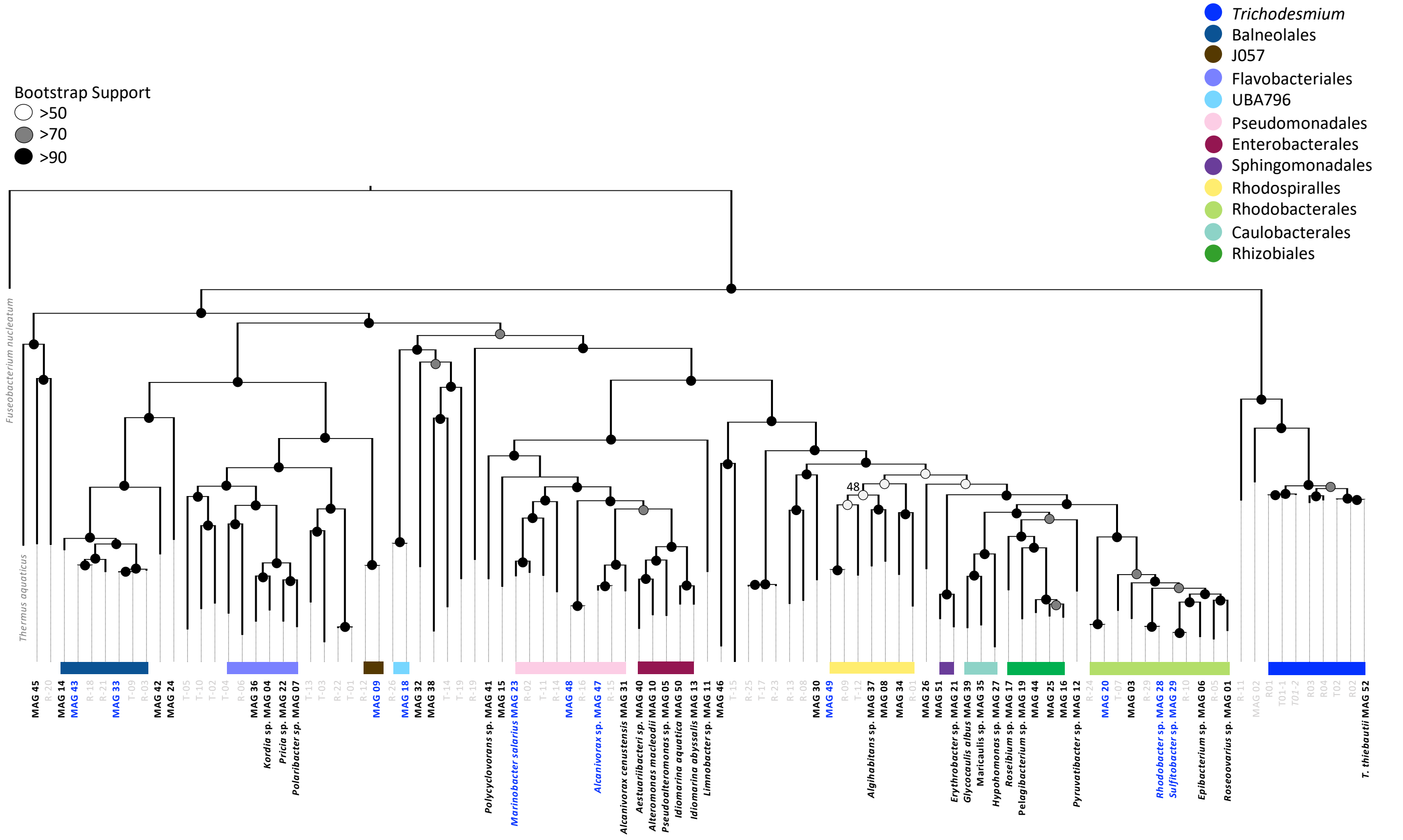


**Supplementary Figure 2. Phylogenetic tree of the 52 MAGs together with MAGs assembled from 3 other Trichodesmium metagenomic datasets from colonies collected in the Red Sea** (38)**, the Pacific** (18, 23) **and the Atlantic** (19) **Oceans.** MAGs from the 3 other datasets are highlighted in grey. MAGs deriving from samples of the Atlantic or Pacific Ocean are listed as T-XX and those from the Red Sea are listed as R-XX. The 11 MAGs that matched to those assembled from previous metagenomic datasets, using a 97.5 % identity cutoff, are highlighted in blue. Known bacterial orders are marked using different colored squares. The tree is rooted by the outgroup *Fuseobacterium nucleatum*. The full taxonomic description of each MAG in this dataset can be found in Supplementary Table 1 and others in Supplementary Table 2.
