# Supplementary figures and images for: Taxonomic distribution of metabolic functions in bacteria associated with Trichodesmium consortia
Source: mSystems. 2023 Nov 2;8(6):e00742-23. doi: 10.1128/msystems.00742-23 (PMC10734445; doi:10.1128/msystems.00742-23)

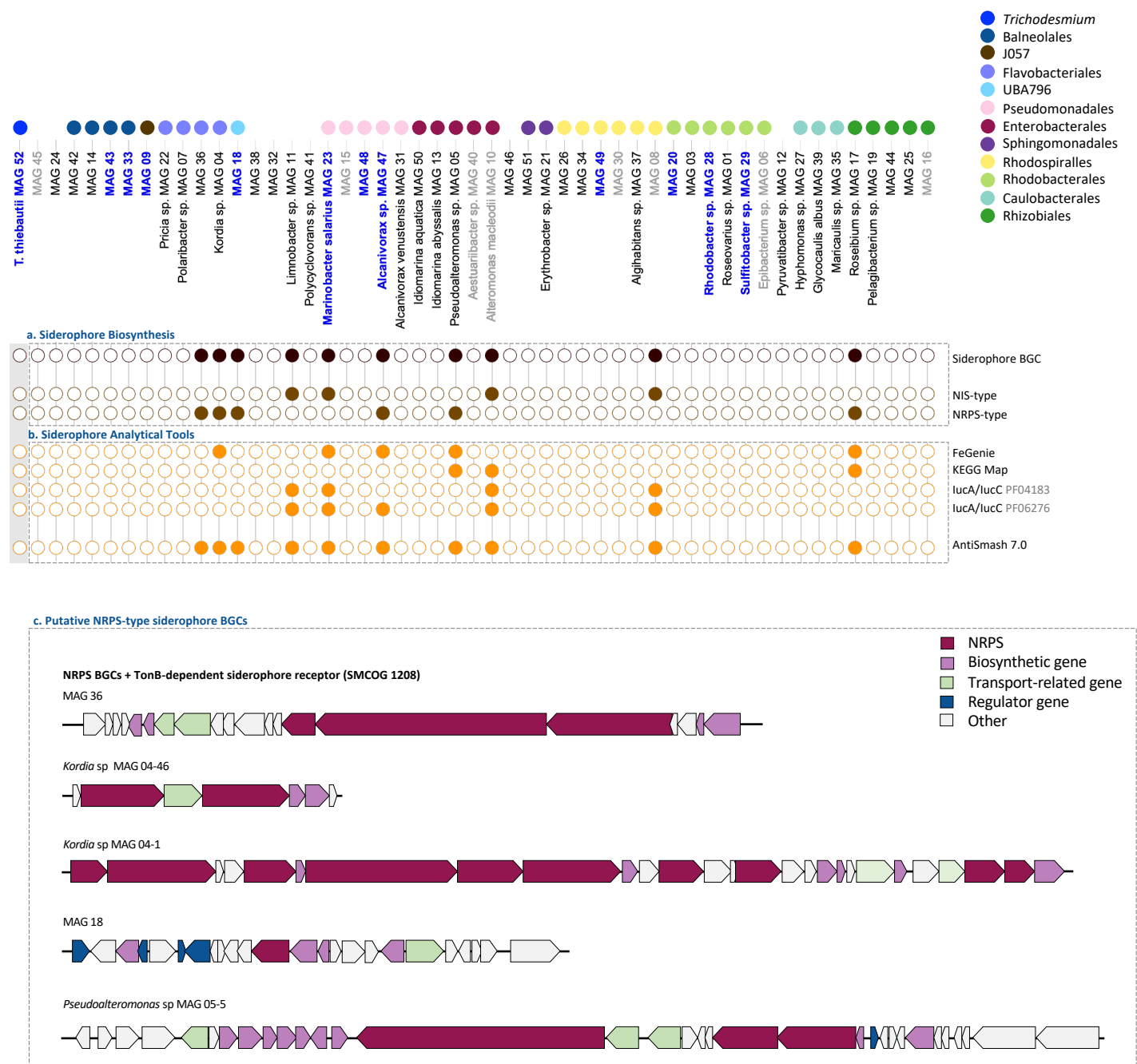

Supplement: Figure S3 — NIS and NRPS-type siderophore biosynthesis pathways identified in MAGs of associated bacteria. [file msystems.00742-23-s0003.pdf]
