# Supplementary material for: Taxonomic distribution of metabolic functions in bacteria associated with Trichodesmium consortia
Source: mSystems. 2023 Nov 2;8(6):e00742-23. doi: 10.1128/msystems.00742-23 (PMC10734445; doi:10.1128/msystems.00742-23)
Supplement: Figure S4 — MAGs were probed for bacterial-interactive and particle-interactive traits. [file msystems.00742-23-s0004.pdf]

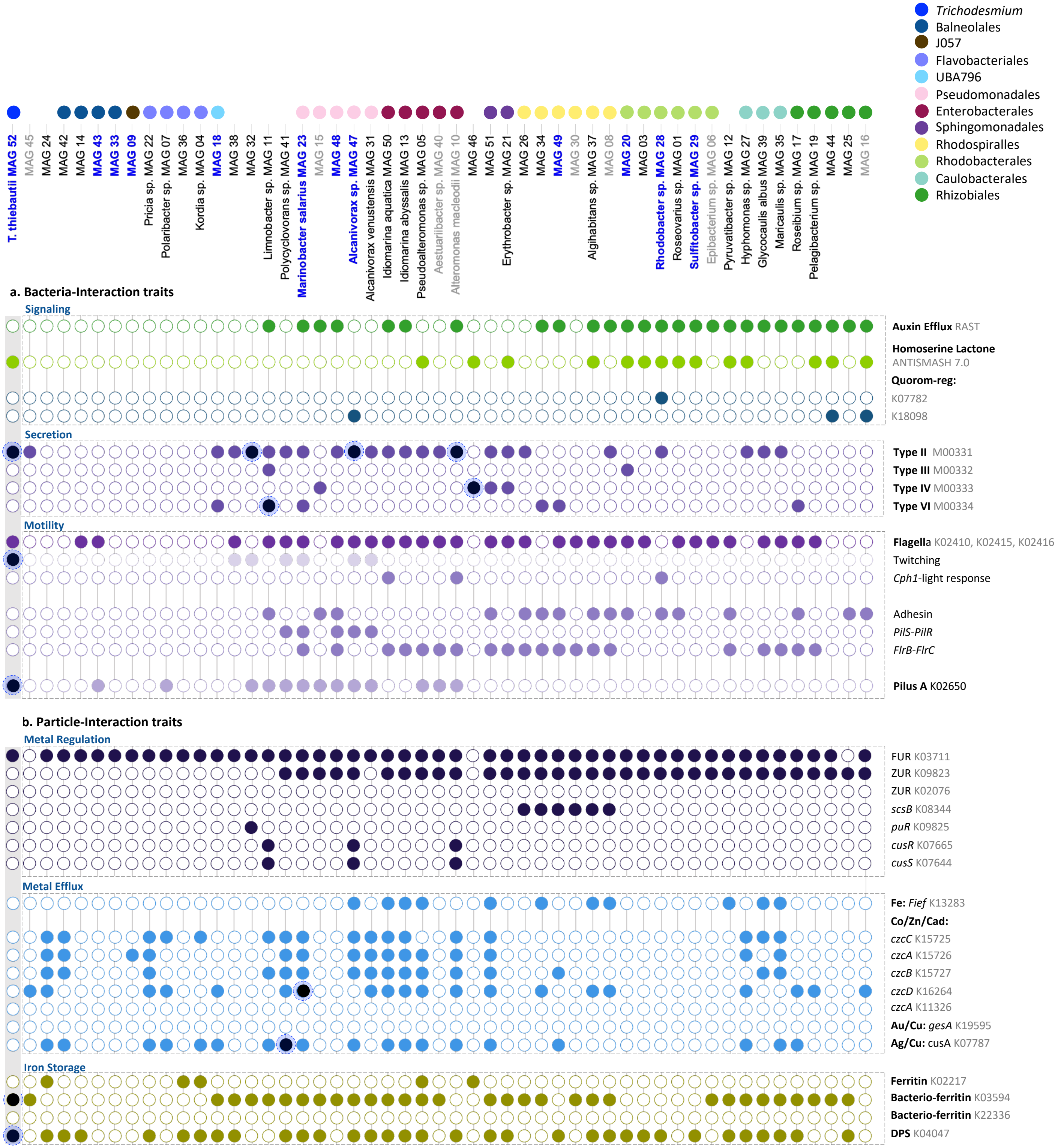

Supplementary Figure 4. (a) MAGs were probed for bacterial-interactive traits (90). MAGs from the phyla Proteobacter, contain the presence of several interactive traits, including quorum sensing, gliding motility, adhesion, cell-to-cell signaling, and antibiotic resistance marker genes. Intriguingly, both the Trichodesmium MAG and several Proteobacter MAGs (e.g. Rhodobacter) contain genes for homoserine lactones (HLs), which are putative quorum sensing molecules. **(b) MAGs were probed for particle-interactive traits** (91**).** In comparison to *T. thiebautii* MAG 52, many MAGs from associated bacteria contained a wide variety of heavy metal efflux systems and metal-transcription factors. While *Trichodesmium*, like most Cyanobacteria, is known to be sensitive to heavy metals, our results indicate that the consortium is attuned towards an environment rich in metals, including particulate dust (see Supplementary Table 4 and material and methods for more details).
